# Unexpected Malignancy in Women Undergoing Surgery for Uterine Fibroids

**DOI:** 10.7759/cureus.104342

**Published:** 2026-02-26

**Authors:** Bandana Bharali, Deepti Goswami, Nita Khurana

**Affiliations:** 1 Gynecology, Maulana Azad Medical College, New Delhi, IND; 2 Pathology, Maulana Azad Medical College, New Delhi, IND

**Keywords:** fibroids, histopathology, leiomyosarcoma, pre operative suspicion, unexpected malignancy

## Abstract

Background: Fibroids are the most common tumors of the uterus and female genital tract. There are several treatment modalities for fibroids, spanning from watchful waiting, medical management, and surgical management, of which surgery continues to be the mainstay of treatment. It has been observed that in a few rare cases, what are presumed to be fibroids preoperatively can reveal malignancy in histopathology, the consequences of which can be grave.

Objective: The primary objective of this study was to estimate the prevalence of unexpected uterine malignancy in women undergoing surgery for uterine fibroids. The secondary objective was to study the clinical characteristics of patients with such unexpected uterine malignancy detected on histopathology.

Methods: We conducted an observational study including all patients who underwent myomectomy or hysterectomy, by any route, for uterine fibroid at the Department of Obstetrics and Gynecology, Maulana Azad Medical College and Lok Nayak Hospital, New Delhi. Any case with preoperative diagnosis of endometrial hyperplasia with atypia or any suspected malignancy was excluded. Data were collected over a period of three years from gynecologic operation theatre records, histopathology reports, and patient files. The prevalence of unexpected uterine malignancy was estimated overall.

Results: A total of 436 women underwent surgery for uterine fibroids in our study; among them, three cases of unexpected malignancy and two cases of smooth muscle tumours of uncertain malignant potential (STUMP) were identified, thus making the prevalence of 0.68% for unexpected uterine malignancy in fibroids.

Conclusion: The absolute risk of unexpected malignancy in women with fibroids is low, but the difficulty in preoperative diagnosis remains a challenge. Hence, patients should be counselled preoperatively about the likelihood of such a finding in the final histopathology.

## Introduction

Uterine fibroids (leiomyoma) are benign monoclonal tumors of smooth muscle cells of myometrium with a varied prevalence, reported to be 4.5-68.6% [1,2]. The prevalence in India was found to be 37.6% and 24% in rural and urban populations, respectively, in women aged 26-55 years [3]. Fibroids are the leading indication of hysterectomy, which remains the most common surgical management [4,5]. Myomectomy is a safe alternative to hysterectomy in women who wish to retain their uterus. It can be carried out by open abdominal route, hysteroscopically, laparoscopically, and via robotic-assisted laparoscopy. Minimally invasive surgery has fewer complications, less postoperative pain, shorter hospital stay, and spares the patient from increased morbidity associated with open procedures [6,7]. However, laparoscopic myomectomy or hysterectomy often requires morcellation of the specimen, which can lead to dissemination of occult malignancy [8,9]. The risk of unexpected malignancy in fibroids is estimated to be one in 352 for any sarcoma and one in 498 for leiomyosarcoma by the FDA in its 2014 statement [8]. In 2017, the FDA updated its assessment by reviewing 23 studies (90,910 women), and the prevalence of uterine sarcoma was one in 305 to one in 360, and the prevalence of leiomyosarcoma was one in 570 to one in 750, which was consistent with its previous statement [10].

Uterine sarcomas are rare tumours of the uterus, accounting for approximately 3-7% of all uterine malignancies [11-13]. Patients with uterine sarcomas have symptoms vaguely similar to those of patients with fibroids. Preoperative diagnostic modalities employed to diagnose uterine sarcomas are not as reliable as those used in the diagnosis of epithelial endometrial malignancies [14,15]. Apart from sarcomas, there is another entity called smooth muscle tumour of uncertain malignant potential (STUMP), which can't be classified equivocally as benign or malignant, but has a less predictable behaviour [16].

Several studies have been done to estimate the incidence of malignancy in presumed uterine fibroids; however, not many studies are available pertaining to Indian data. Hence, we aimed to find the prevalence of unexpected uterine malignancy in women undergoing surgery at our institution for presumed uterine fibroids and to study the clinical characteristics of such patients.

## Materials and methods

We conducted a retrospective observational study on patients who underwent myomectomy or hysterectomy for uterine fibroids at the Department of Obstetrics and Gynecology, Maulana Azad Medical College and Lok Nayak Hospital, New Delhi, from January 2018 to December 2021. All patients who underwent myomectomy or hysterectomy, by any route, for uterine fibroids were included. Any case with preoperative diagnosis of endometrial hyperplasia with atypia or any suspected malignancy was excluded from the study. Data pertaining to demographic details, symptomatology, preoperative evaluation, operative findings, and histopathology were collected from gynaecologic operation theatre records, histopathology reports, and case files. The histopathology reports and files of the patients found to have malignancy were studied in detail.

The collected data were transformed into variables, coded, and entered into Microsoft Excel (Redmond, WA, USA). Statistical testing was conducted with SPSS Statistics version 25.0 (IBM Corp., Armonk, NY, USA). Continuous variables were presented as mean +/- SD for normally distributed data or median (IQR) for non-normally distributed data. Categorical variables were expressed as frequencies and percentages.

We primarily aimed to find the occurrence of unexpected uterine malignancy in women undergoing surgery for uterine fibroids. As a secondary outcome, we aimed to study the clinical characteristics of patients with such unexpected uterine malignancy detected on histopathology.

The prevalence of unexpected uterine malignancy is expressed as a rate or percentage per 100 hysterectomies or myomectomies.

## Results

The present study included 436 women who underwent surgery for presumed leiomyomas at the Department of Obstetrics and Gynaecology, Lok Nayak Hospital.

Abnormal uterine bleeding was the most common presenting complaint (80.9%), followed by pelvic mass (46.5%) and pelvic pain (40.3%). Most patients harboured multiple complaints. The mean age of presentation in our study was 40.5+/-7.5 years; most were premenopausal. Around 17% were nulliparous.

Ultrasound documenting the number, location, size, and sonographic features of fibroids was done for all the patients. Suspicious features like heterogeneous echogenicity, irregular contour, and increased vascularity on Doppler were not reported in any. MRI was done in a selected few cases for fibroid mapping or confirmation of diagnosis. Site, size, and number of fibroids could be delineated by ultrasound in most cases, along with degenerative changes. However, no abnormality apart from fibroid was suspected in either case of endometrial stromal sarcoma, STUMP, or mixed Mullerian tumour by ultrasound or MRI.

Hysterectomy was the most common procedure performed. Three hundred nine patients (70.8% of our study subjects) underwent hysterectomy. Laparotomy was done most commonly. Laparoscopic hysterectomy was carried out in seven patients, including laparoscopy-assisted vaginal hysterectomy in two. Myomectomy was done in 125 patients (28.6%). It was done by laparotomic approach in most of the cases. Laparoscopic myomectomy was done in two patients. A morcellator was used in the cases of laparoscopic myomectomy but not for laparoscopic hysterectomy.

**Figure 1:**
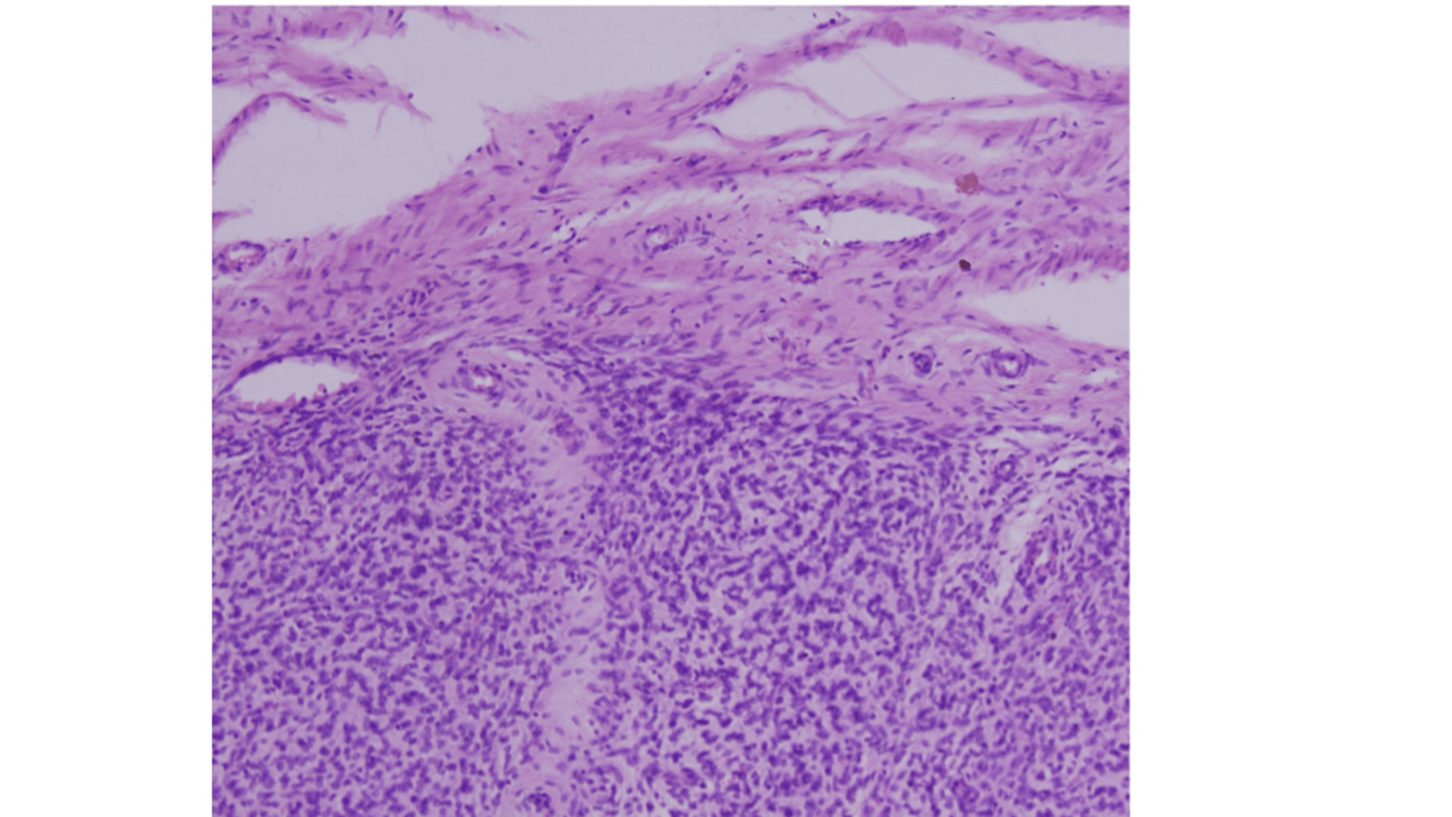
Histopathological image of low-grade endometrial stroma sarcoma (Haematoxylin & Eosin, 400x) of 38-year-old patient presenting with heavy menstrual bleeding.

Among the 436 cases studied by us, we found three cases of unexpected malignancy: two low-grade endometrial stromal sarcomas and one case of mixed Mullerian tumour, thus making the prevalence of unexpected malignancy in patients operated for presumed fibroids 0.68% (1 in 145). There were two cases of STUMP. We didn’t find any case of unexpected leiomyosarcoma. The three cases of unexpected malignancy are described in Table 1, and a histopathological image of low-grade endometrial stromal sarcoma is shown in Figure 1. The prevalence observed by us was higher than that reported by the FDA in 2014, i.e., one in 352 for any sarcoma and one in 498 for leiomyosarcoma [8]. The committee opinion by the American College of Obstetricians and Gynecologists (ACOG) in 2019 found that the risk of unexpected leiomyosarcoma was reported to range from less than one in 770 to one in 10,000 surgeries for presumed leiomyoma [9].

**Table 1:** Clinical characters of the cases with unexpected malignancy ER: estrogen receptor, PR: progesterone receptor

	Patient A	Patient B	Patient C
Diagnosis	Low-grade endometrial stromal sarcoma	Low-grade endometrial stromal sarcoma	Mixed Mullerian tumour
Age	38	34	42
Menopausal status	Premenopausal	Premenopausal	Premenopausal
Symptoms	Heavy menstrual bleeding (Required blood transfusion)	Heavy menstrual bleeding, abdominal mass	Abdominal pain, heavy menstrual bleeding, abdominal mass
Clinical examination	Abdominal examination normal. Mass of size ~8cm felt in left side of pelvis, not separate from uterus on per vaginal examination.	A large abdominopelvic mass of 24-week size palpable, firm in consistency, mobile, non-tender.	Mass of 24-week size palpable in suprapubic region with smooth margins, restricted mobility.
Imaging	USG: 5x2 cm intramural fibroid on anterior and left myometrium. MRI: Multiple variable-sized fibroids with degeneration in anterior and posterior myometrium. 2.6x1 cm size endometrial polyp.	USG: 15.7 x 11 cm intramural fibroid in right posterior uterine wall. MRI: 18x 13x 12 cm fibroid in fundal region of uterus indenting endometrium.	USG: 6.6 x 6.8 cm intramural fibroid in anterior uterine wall.
Preoperative endometrial histopathology	Record not available.	Record not available.	Late proliferative endometrium.
Procedure	Total abdominal hysterectomy.	Planned for myomectomy. Converted to total abdominal hysterectomy in view of suspicious intraoperative findings.	Total abdominal hysterectomy with bilateral salpingectomy.
Uterine size	6-8 weeks	24 weeks	18 weeks
Number of fibroids	2	3	8
Size of largest fibroid (in greatest dimension)	6 cm	10 cm	10 cm
Histopathology features	Grey-white area in endometrial cavity, 2 submucosal fibroids seen, necrosis+, Sections from grey white area: features of low-grade endometrial stromal sarcoma	Endometrial cavity pushed to one side by growth which seems to be arising intramurally, measuring 14x 13x 8 cm. Growth appears brown to yellow, variegated. Exophytic papillary projections seen at superior aspect of growth. Histologic type- endometrial stromal sarcoma, low grade, more than 50% myometrial invasion. Cells positive for CD10, negative for ER, PR.	4 intramural, 1 submucosal, 2 subserosal fibroids 3.5x3 cm fibrofatty tissue, sections show features of mixed Mullerian tumour, necrosis +, Cells positive for cytokeratin and vimentin

## Discussion

Fibroids are the most common tumour of the uterus and female genital tract, and the leading cause of hysterectomy. There is a rare possibility of detecting unexpected malignancy in a uterine mass presumed to be a fibroid, which significantly affects the management and outcome. This forms the crux of our study. Uterine sarcomas are relatively rare, potentially aggressive gynecologic malignancies. Sarcomas have been challenging to diagnose preoperatively due to limitations in clinical and radiological predictors. While fibroids are common in the reproductive age group, the average age of diagnosis of sarcomas is 55 years. The mean age of presentation of our study subjects was 40.3 +/- 7.5 years. The three cases of unexpected malignancy in our study (two cases of low-grade endometrial stromal sarcoma and one case of mixed Mullerian tumour) were 34, 38, and 42 years old, respectively. A population-based study by Mao et al. emphasized the relation between age and prevalence of sarcoma among patients with leiomyoma. In their study, the prevalence was the lowest in women younger than 50 years of age (0.8-1.3 per 1000), whereas this rate was the highest in women older than 60 years of age (3.6- 15.3 per 1000) [17]. Similar findings were described in studies done by Rey et al., Kho et al., and Ruengkhachorn et al. [18-20].

Both cases diagnosed as endometrial stromal sarcoma in our study had presented with heavy menstrual bleeding, and one of them also had a palpable abdominopelvic mass corresponding to a 24-week-sized uterus. The patient with mixed Mullerian tumour had presented with heavy menstrual bleeding, abdominal pain, and a palpable uterus of 24-week size. Heavy menstrual bleeding has been reported to be the most common symptom in previously done studies by Cao et al., Damasco et al., Ruengkhachorn et al., Paul et al., and Mettler et al. [20-24].

Preoperative imaging detected no unanticipated findings in either of the cases of malignancy, nor did endometrial sampling raise any suspicion. Several imaging modalities have been described for preoperative evaluation of uterine sarcomas, like computed tomography, Doppler USG, and MRI, but the accurate diagnosis remains elusive.

Intraoperative suspicion of a non-benign nature of growth was found in one of the cases of endometrial stromal sarcoma. The 34-year-old multiparous patient was taken up for myomectomy, however during dissection, the endometrial cavity was breached and an irregular polypoidal growth was seen in the uterine cavity. The specimen was sent for frozen section analysis, where the possibility of malignancy could not be ruled out, and the procedure was converted to total abdominal hysterectomy in view of such findings. Final histopathological report revealed low-grade endometrial stromal sarcoma.

The risk of unexpected malignancy in women with fibroids in our study came out to be 0.68% (one in 145). The risk is higher compared to that determined by the FDA, which can be explained by the smaller sample size in our study. It is important to be aware of this risk for various reasons, including counseling regarding the risks and benefits of the surgical approach, appropriate surgical planning, and avoidance of iatrogenic complications like the dissemination of occult malignancy by use of a power morcellator.

The drawback of our study is the retrospective nature, with its inherent problems like missing data and the relatively small sample size. The strength of our study lies in the fact that it has been carried out in a tertiary care center and is the second largest study on this subject carried out in India after Paul et al., who published their retrospective study of patients over 10 years ago in 2015 [21].

## Conclusions

The prevalence of unexpected uterine malignancy in women undergoing surgery for presumed uterine fibroids is 0.68% in our study. Preoperative imaging didn’t help in identification due to a similar appearance to fibroids on USG and MRI, nor did preoperative histopathological evaluation of the endometrium.

Hence, even though the absolute risk of unexpected malignancy in fibroids is less, the possible risk should be kept in mind, and preoperative counselling should include information about such a risk.
